# Changes in Total Energy, Nutrients and Food Group Intake among Children and Adolescents during the COVID-19 Pandemic—Results of the DONALD Study

**DOI:** 10.3390/nu14020297

**Published:** 2022-01-11

**Authors:** Ines Perrar, Ute Alexy, Nicole Jankovic

**Affiliations:** 1 Institute of Nutritional and Food Sciences-Nutritional Epidemiology, University of Bonn, Friedrich-Hirzebruch-Allee 7, 53115 Bonn, Germany; 2 Institute of Nutritional and Food Sciences-Nutritional Epidemiology, University of Bonn, DONALD Study, Heinstück 11, 44225 Dortmund, Germany; alexy@uni-bonn.de (U.A.); njankovi@uni-bonn.de (N.J.)

**Keywords:** COVID-19, nutrition, children, adolescents

## Abstract

The COVID-19 pandemic may have changed the habitual lifestyles of children and adolescents, in particular, due to the closure of kindergartens and schools. To investigate the impact of the pandemic on nutrients and food intake of children and adolescents in Germany, we analyzed repeated 3-day weighed dietary records from 108 participants (3–18 years; females: *n* = 45, males: *n* = 63) of the Dortmund Nutritional and Anthropometric Longitudinally Designed (DONALD) study. Polynomial mixed-effects regression models were used to identify prospective changes in dietary intake (total energy (TEI), carbohydrates, fat, protein, free sugar, ultra-processed foods, fruits and vegetables, sugar sweetened beverages and juices) before and during the first months of the COVID-19 pandemic. For the current analysis, we have chosen the first months of the pandemic (March 2020–August 2020), as this was the period with the most restrictions in Germany so far (kindergarten, school and restaurant closures; contact and outdoor activity restrictions). No significant changes in either the selected nutrients or food groups were observed. However, children and adolescents recorded a significantly lower TEI during the pandemic (β = −109.65, *p* = 0.0062). Results remained significant after the exclusion of participants with under-reported records (β = −95.77, *p* = 0.0063). While macronutrient intake did not change, descriptive data indicate a non-significant decrease in sugar sweetened beverages and ultra-processed foods intake. We suggest that children and adolescents from high socioeconomic families may have adapted lifestyle changes during the pandemic.

## 1. Introduction

The COVID-19 outbreak starting in the year 2020 in Wuhan, China, has challenged the habitual lifestyle of people all over the world. In many countries, human contact was limited by governments, to minimize the spread of the virus. In Germany, the federal states proclaimed contact restrictions measures in March 2020 [1,2], after the World Health Organization (WHO) declared COVID-19 to a global pandemic [3]. Only supermarkets and drugstores were allowed to open for essential needs and restaurants were allowed to sell take away food only. Additionally, due to social distancing and the regulation of the governments in Germany, families had to spend more time at home and were requested to reduce meetings with friends or family members, who do not live in their own household. However, one of the biggest challenges for families during the lockdown in 2020 was the lack of institutional childcare and education. Many parents had to organize childcare and/or schooling of their children at home. Among others, this development may have led to a new food and eating environment, in particular, if children usually had breakfast and/or lunch in kindergarten or school.

Some studies suggest changes in lifestyle (including diet) among children and adolescents during the COVID-19 pandemic [4,5,6]. However, most studies do not include objective control data before the pandemic, and therefore, are based on subjective estimations by the participants themselves. Only one study investigated longitudinal changes in diet among children and adolescents in Italy based on telephone interviews, with just 41 obese participants [7]. Therefore, the investigation of changes in diet quality among healthy children and adolescents based on data from longitudinal studies is needed.

Several parameters are used as descriptors of diet quality in nutritional studies, e.g., energy and macronutrient [8,9], free sugar intake [10,11] and the intake of selected food groups, e.g., ultra-processed foods [12]. High intakes of free sugars, sugar sweetened beverages (SSB) [11], or ultra-processed foods (UPF) [12] are discussed to contribute to the development of various diseases, such as overweight or obesity, cardiovascular diseases, or metabolic syndrome. In contrast, high intakes of other parameters, such as fruits and vegetables, legumes and grains are associated with health benefits, e.g., a protective impact on the development of overweight, obesity, or cardiovascular diseases [13]. Such changes in dietary quality during childhood and adolescence may track into adulthood [14]. Furthermore, in particular, primary school age and adolescence are seen as “critical periods” for the development of diseases, e.g., obesity or type 2 diabetes, in later life [15,16]. Therefore, pandemic-related changes in dietary intake among children and adolescents, especially towards a poorer diet quality, should be identified.

For that purpose, we compared dietary intake among children and adolescents aged 3–18 years during the pandemic between March 2020 and August 2020 with pre-pandemic dietary intake, using data from 108 3-day weighed dietary records from the Dortmund Nutritional and Anthropometric Longitudinally Designed (DONALD) study.

## 2. Materials and Methods

### 2.1. Study Design and Sample

The DONALD study was designed over 35 years ago as an ongoing cohort study to collect data on diet, growth, development and metabolism of healthy children and adolescents in Dortmund, Germany and surrounding areas. Since 1985, 35–40 infants have been newly recruited every year. Eligible to participate are healthy German infants (i.e., infants free of diseases affecting growth and/or dietary intake). Furthermore, parents have to be willing to participate in a long-term study. Since all study documents, e.g., questionnaires and dietary record descriptions are written in German, at least one parent has to have sufficient knowledge of the German language to understand the contents and provide the appropriate information. Examinations in DONALD usually start at the age of 3 months. Participants return afterward for three more visits in the first year, two in the second year and thereafter annually until young adulthood. Annual examinations include 3-day weighed dietary records, anthropometric measurements, collection of 24-h urine samples (starting at age 3–4 years), interviews on lifestyle and medical examinations. Parental examinations (anthropometric measurements, lifestyle interviews) take place every four years. Further details about the study have been described elsewhere [17,18]. The DONALD study was approved by the Ethics Committee of the University of Bonn according to the guidelines of the Declaration of Helsinki. All examinations are performed with parental and, from the age of 16 years onwards, on children’s written consent.

In Germany, the pandemic lockdown started in March 2020 [1,2] and was eased in May 2020 [19,20]. However, in North Rhine-Westphalia normal operations in schools and kindergartens or other childcare institutions were still not possible due to high incidence rates until summer break, so that children were mainly supervised at home. Therefore, we chose the time period between March and August 2020 for the current analyses.

Since the participants are usually invited for the examinations at the same time each year (in the weeks around the participants’ birthdays), approx. 200 participants between the ages of 3 and 18 were contacted during March and August 2020, as in previous years. However, medical and anthropometric examinations at the study center were prohibited during these months. Participants were instead asked to complete questionnaires at home as well as to collect a 3-day weighing dietary record and 24 h-urine as usual. Unfortunately, the response rate of the participants was lower during the pandemic. While in the years before the pandemic, more than 150 participants came to the study center for examination between March and August, in 2020 only 119 participants provided anthropometric data at this time of the year. In addition, usually around 130 dietary records were collected during this period before the pandemic, while only 110 dietary records were collected in these months in 2020.

Hence, for the current analyses we use data from all participants in the DONALD study (*n* = 108; 3–18 years), who had collected one 3-day weighed dietary record during the first months of the pandemic (from 14th of March in 2020 until 11th of August in 2020) as well as one record one or two years prior to the COVID-19 pandemic (*n* = 101 in 2019; *n* = 7 in 2018).

### 2.2. Dietary Assessment

Dietary data were collected using the same method before and during the COVID-19 pandemic. 3-day weighed dietary records were conducted before and during the pandemic, respectively. Electronic food scales (±1 g) are used to weigh all foods and beverages consumed by the participant, as well as leftovers by the parents or by the older DONALD study participants themselves over 3 consecutive days. Participants can choose the day of the beginning of dietary recording. When exact weighing is not possible, participants are allowed to use household measures (e.g., spoons, cups) for semi-quantitative recording. Information on recipes, i.e., ingredients and preparation and on the types and brands of food items consumed is also requested. Medication and dietary supplement use are also recorded but were not considered for the current analyses. A trained dietitian checks the dietary records for accuracy and completeness prior to adding the new data to our database. Subsequently, energy and nutrition intakes are calculated using our continuously updated in-house nutrient database LEBTAB [21]. The composition of staple foods is based on the German food composition tables BLS 3.02 (https://www.blsdb.de/; last accessed on 6 January 2022). Energy and nutrient contents of commercial food products, i.e., processed foods and ready-to-eat meals or snack foods are estimated by recipe simulation using labeled ingredients and nutrient contents.

In the current analyses intake of total energy intake (TEI; in kcal), nutrient intake (fat, protein, carbohydrates, free sugar ([FS]) in the percentage of total daily energy intake (%E)) as well as the intake of different food groups (fruit & vegetables, SSB, juices and UPF in g/1000 kcal) were calculated as individual means from 3 days of recording. These variables were investigated as outcome variables of the statistical models. For free sugar, we expanded the definition by the WHO (“All monosaccharides and disaccharides added to foods by the manufacturer, cook, or consumer, plus sugars naturally present in honey, syrups, and fruit juices”) [11] as suggested by SACN [22,23]: Since “food subject to blending, pulping, or macerating which breaks down the cellular structure should also be considered as containing free sugars” [23], sugars from vegetable juices, juice spritzers and smoothies were also considered as FS in the present evaluation. UPF was categorized according to the NOVA classification of Monteiro et al. 2010 [12,24]. A list of foods, which are used to calculate the intake of the investigated food groups is shown in Table 1.

### 2.3. Childcare Characteristics during the Pandemic

In June 2020, parents or older children were asked to answer a short questionnaire regarding children’s daycare during the pandemic. First, the type of childcare during the pandemic was asked (“school”, “kindergarten” or “others (e.g., by family members)”). In Germany, it was possible for parents from system-relevant professions, such as doctors or nurses to use a so-called “emergency childcare” for their children while kindergarten and schools were officially closed. Therefore, the use of childcare during the pandemic was also queried: “Did you used the “emergency childcare“ during pandemic? (yes/no)”. In addition, we asked if one or both parents were worked in a home office during the pandemic.

### 2.4. Assessment of Potential Confounding Factors

For the current analysis, the following characteristics were considered as potential confounding factors: sex (boy/girl), age (years), overweight status of the participant (yes/no), number of weekdays per 3-day record (1/2/3), the season of the 3-day record (summer/autumn/winter/spring), maternal overweight (yes/no), as well as socioeconomic factors (high maternal educational status (yes/no), maternal employment (yes/no)).

During the first month of the pandemic, it was prohibited to carry out any anthropometric measurements at the DONALD study center. Before pandemic, height and weight are measured by trained nurses according to standard procedures with the participants dressed in underwear only and barefoot. From the age of 2 years onwards, standing height is measured to the nearest 0.1 cm using a digital stadiometer (Harpenden, Crymych, UK). Bodyweight is measured to the nearest 100 g using an electronic scale (Seca 753E; Seca Weighing and Measuring System) [18]. During the lockdown in 2020, participants were asked to self-report their measured body height and weight at home.

Body mass index (BMI, kg/m^2^) was calculated as the body weight (kg) divided by the square of the body height (m^2^). Overweight was defined according to International Obesity Task Force’s (IOTF) BMI cut-off values for children and adolescents [25,26]. Maternal body weight and height are measured with the same equipment as for the participants. Maternal overweight was defined as a BMI ≥ 25 kg/m^2^. Socioeconomic factors, i.e., high maternal educational status (<12 years of schooling) as well as maternal employment were inquired with a standardized questionnaire. Missing values occurred for maternal overweight (*n* = 7), only. These values were imputed by the respective median of the total sample.

### 2.5. Statistical Analysis

The statistical analyses of the current study were performed using SAS^®^ procedures (version 9.4; Cary, NC, USA). The significance level was set at *p* < 0.05. Characteristics (Pre-pandemic and pandemic) of the participants are presented as mean ± SD (for normally distributed variables) medians with their interquartile range (for non-normally distributed variables) or frequencies and percentages (Table 2 and Table 3).

Linear mixed effects regression models (PROC MIXED in SAS^®^ Cary, NC, USA) including both fixed and random effects were used to investigate the impact of the pandemic (predictor) on repeated dietary intake variables (outcome). Since statistic tests indicated no significant interactions between sex and the predictor in each statistical model, data from girls and boys were not stratified for analysis. Random or repeated statements were included in the statistic models if they improved the fit statistics [Akaike information criterion (AIC)] by more than two points or significantly predicted the respective outcome [30,31]. A repeated statement was considered to account for the lack of independence between repeated measures from the same person. Random effects were considered to allow variation between individuals and families with respect to the initial level (intercept). The AIC was also used to select the covariance structure that best describes the variance and covariance of the initial level as wells as random effects and the covariance structure that best describes the correlated nature of the repeated measurements.

The crude models include outcome (dietary variable), predictor (pandemic (pandemic/pre-pandemic)) and are adjusted for sex (male/female). Further covariates that were considered in the final models either (1) modified regression coefficients in the basic models by 10% (2), had a significant and independent association with the outcome variable, or (3) led to an improvement of the AIC by more than two points [30]. Following this procedure, all adjusted models were further adjusted for age (years). In addition, some covariates (season (summer/autumn/winter/spring), maternal employment (yes/no), overweight status of the participant (yes/no), high maternal education (yes/no)) were relevant for individual models, only. The description of all models is provided in Table 4 and Table S1. Pre-pandemic data from the same participants were used as a reference in all statistical models. The final models were tested for heteroscedasticity and normal distribution of the residuals.

Sensitivity analyses were performed excluding participants with under-reported records (Table S1). Records were considered as under-reported when the total energy intake (TEI) was inadequate in relation to the estimated basal metabolic rate (BMR) (according to age- and sex-specific equations of Schofield [32]), using paediatric cut-offs [27].

## 3. Results

Characteristics of the participants, before and during the pandemic are shown in Table 2. Anthropometric and socioeconomic characteristics of the participants and their families reflect the high socioeconomic status (SES) of DONALD participants (Table 2). The descriptive analyses (Table 2) indicate dietary changes in the mean intake of TEI (−0.85%), UPF (−2.0%) and especially in median intake of SSB (−100%) and juices (+30.4%).

Childcare characteristics are shown in Table 3. The childcare questionnaire was delivered from 65% of the participants (*n* = 71) during the pandemic. Most children were supervised at home due to school or kindergarten closures by their parents and the majority of parents worked from home (Table 3).

The statistical comparison of dietary characteristics results in a significantly lower TEI among children and adolescents during the pandemic (β = −109.65, *p* = 0.0062; Table 4). Intakes of carbohydrates, fat, proteins, free sugar and fruits and vegetables did not statistically differ before and during the pandemic. Although β-estimates suggest relevant changes in intake, no significant results were observed for changes in UPF, SSB and juice intake among the present sample.

Among 17 (15.7%) participants, dietary records were identified as under-reported. After excluding under-reported records in the sensitivity analyses, similar results were observed and changes in TEI remain significant (β = −95.77, *p* = 0.0063; Table S1).

As many participants had not completed the questionnaire on physical activity, this variable could not be used as a potential covariate in the main analysis. In total, data from 73 participants were available. However, the descriptive analysis (Table 2) showed no differences between the participants before and during the pandemic. Sensitivity analyses (*n* = 73) in which physical activity was tested as an additional covariate also showed similar results (data not shown).

## 4. Discussion

In the present evaluation, we investigated changes in dietary intake (TEI, selected nutrients and food groups) among children and adolescents in Germany during the COVID-19 pandemic (14th March–11th August 2020), using data of an ongoing cohort study. We observed that TEI of children and adolescents was significantly lower during the pandemic, while nutrient and food group intake did not change.

The observed results are encouraging as experts are concerned that the pandemic will promote unhealthy lifestyle patterns. A trend towards undesirable food choices together with a reduction in physical activity might promote the development of obesity [4,5,6]. As our data did not allow us to attribute TEI changes to concurrent changes in macronutrients or food group intake, we assume a reduction in the total amount of food consumed. However, the descriptive analysis, as well as the β-estimates, indicate a decline in UPF and SSB. We can only speculate about the underlying causes. It is possible, that in times of the COVID-19 pandemic, a healthy lifestyle was adhered to, to strengthen a healthy immune system, and therefore, avoid a COVID-19 infection. The descriptive increase, as well as the observed β-estimates for juice intake in the present study, could support this, as juices probably reflect the recognition of fruit juice as a “healthy” beverage in Germany, even though pure fruit juices have a similar sugar and energy content as SSB [33,34].

Furthermore, it is discussed whether children can naturally compensate for their TEI according to their caloric intake and their energy expenditure. Several studies have shown the ability to compensate for a high-caloric snack or meal at a following meal or during the day, especially among younger children [35,36] and regardless of body composition [37]. This natural ability could also benefit dietary intake among children and young people during the pandemic when physical activity was reduced because, for example, sports clubs were not allowed to offer training.

To our knowledge, this is the first cohort study, which analysed prospective data on changes in energy, selected nutrient and food group intake during the COVID-19 pandemic among children and adolescents in Germany. Other surveys give an indication of possible changes among children and adolescents during the pandemic. In a representative German online survey, families with children and adolescents (1–14 years) were questioned about their own estimates of changes in their habitual diet during the pandemic [4]. The observed results indicate that healthier habits, such as cooking at home or the consumption of fruit and vegetables increased but were not validated by statistical tests. Conversely, to our study, the survey also showed an increase in unhealthy food choices, such as an increase in the intake of salty and sweet snacks, pizza and pasta, or soft drinks. Based on their findings, the authors suggest that changes towards a healthier diet are more likely to be observed among families with high socioeconomic status [4]. This is supported by the present results of the DONALD study. Families participating in the DONALD study mainly have a high socioeconomic status (Table 2; [18]), which could be a possible reason why the COVID-19 pandemic did not lead to poorer diet quality among participants. In contrast, the authors suggest that unhealthy habits are more likely to be seen among children with low socioeconomic status, and therefore, propose a “disturbing aggravation of socioeconomic disparity”, with a higher risk of body weight gain among children and adolescents with low socioeconomic status [4].

Recent systematic reviews summarized results of worldwide studies regarding the impact of pandemic confinements or lockdown measures on diet and body weight among children, adolescents and adults [5,6]. The authors mentioned an increase in snack frequency and intake of UPF as well as both an increase and decrease of fruits, vegetable and fresh food intake. In addition, the weight gain of participants depending on their socioeconomic status was reported. However, all data based on online surveys and no cohort studies with pre-pandemic measurements were available to include. Furthermore, results include also data from adults and no studies from Germany were included. It is difficult to compare study results worldwide, as different lockdown measures may have affected people differently. In addition, lifestyles may also differ due to the observed season [38] in different parts of the world.

Some limitations of the present work need to be discussed. As mentioned before, the generalizability of our results is limited due to the high socioeconomic status of the DONALD cohort. Nevertheless, energy, nutrient and food group intake data are similar to representative German nutrition surveys [39,40]. A further limitation of the present evaluation is the small number of participants. Although over 1700 participants were recruited in the DONALD Study, the selection criteria for this evaluation reduced the number of data, in particular, as we kept the time period small to avoid seasonal differences. The low response rate further reduced the sample size. In addition, although study participants are asked to complete a dietary record annually, some study participants do not follow this request every year. Furthermore, no anthropometric measurements were allowed at the study centre during the pandemic. However, BMI and overweight status were similar before and during the pandemic in the present analyses.

Although our study sample for the current analyses is small (108 participants and 216 observations), other longitudinal studies that investigated dietary changes during the pandemic had even smaller study samples [7,41].

Overall, SSB intake among participants in the present study is small. Therefore, residuals of SSB intake were not normally distributed. In addition, no normal distribution was obtained when transforming the data, as a large proportion of participants did not consume SSB within the observed 3 days. Due to the small number, this also applies to the intake of juices, since not all participants consumed juices during the record days. Therefore, these results should be treated with caution.

Even though 3-day weighed dietary records have some advantages compared to, e.g., online questionnaires [42], they are also based on participants’ self-report. Self-reported data may be prone to bias due to underreporting (e.g., underreporting of SSB) [43]. However, our sensitivity analyses, excluding underreported records from the analyses, do not support the notion of bias from underreporting.

The main strength of the present evaluation is the longitudinal design of the DONALD study, which allows the comparison of dietary intake before and during the COVID-19 pandemic among the same participants. A further strength of the study is the use of 3-day weighed dietary records, which have been validated using biomarkers from 24 h urine [44]. In addition, the continuously updated in-house nutrient database LEBTAB allows the consideration of brand-specific energy and nutrient content in commercial products as well as to identify UPF [21]. Finally, due to the extensive data collection in the DONALD study, many covariates could be considered as potential confounding factors.

## 5. Conclusions

In summary, the COVID-19 pandemic has only slightly changed the dietary intake of German children and adolescents in the DONALD study. These observations are encouraging, as the COVID-19 pandemic has not promoted unhealthier dietary habits at least among participants with high socioeconomic status. It would be of scientific interest if any dietary changes can be observed among children and adolescents of low socioeconomic status in Germany, as the pandemic may have increased socioeconomic disparity. In addition, it is questionable whether the observed change in TEI among DONALD participants remains even after the COVID-19 pandemic and thus, represents a long-term change. Therefore, the examination of long-term trends over the entire pandemic would be desirable in the future.

## Figures and Tables

**Table 1:** Classifications of the food groups.

Food Groups	Components
Ultra-processed foods (UPF) ^1^	Ultra-processed dairy (e.g., processed cheese, milk desserts, milkshake, dairy powder, instant milk beverages), ready to eat egg meals (e.g., pancakes), Sugary food and sweets (e.g., syrup, sweet spreads, sweets and marshmallows, chocolate and bars, ice cream, jelly desserts, sweet sauces, sweet baking ingredients (e.g., marzipan)); sweet bread and cakes (incl. baking mixtures); salty snacks; ready to eat cereals and mueslis; ultra-processed meat and fish (e.g., sausages, meat/fish salad, breaded meat/fish); vegetarian/vegan meat/fish substitutes, vegetarian/vegan spreads; vegan milks substitutes with flavor, vegan cheese; potato products (e.g., French fries, croquettes, instant potato dumplings), wrapped bread; ready to bake doughs; filled pasta, e.g., tortellini; ready to eat meals; instant/ready to eat soups, sauces and dressings; formula and baby food; sugar sweetened beverages (e.g., sweetened fruit juice drinks and nectars, soft drinks/sodas, sweetened teas and waters, instant beverages, sweetened sport drinks)
Fruits and vegetables	Fresh, frozen, canned and dried fruits and vegetables
Sugar sweetened beverages	Sweetened fruit juice drinks and nectars, soft drinks/sodas, sweetened teas and waters, instant beverages (except dairy drinks), sweetened sport drinks
Juices	Fruits and vegetable juices, juice spritzers and smoothies

^1^ Classifications of ultra-processed foods (UPF) according to the NOVA classification of Monteiro et al. 2010 [12,24].

**Table 2:** Pre-pandemic and pandemic characteristics of the DONALD participants (3–18 years; males: *n* = 63; females: *n* = 45): dietary, anthropometric and physical activity data as well as socioeconomic factors.

	Pre-Pandemic ^1^	Pandemic ^2^	Changes (%)
Females/males [%]	45 (42)/63 (58)		-
Age [years]	10.3 ± 4.8	11.3 ± 4.8	+9.7
Dietary data			
TEI [kcal/day]	1757 ± 566	1742 ± 489	−0.85
Fat [%E]	34.7 ± 5.3	35.3 ± 6.2)	+1.7
Protein [%E]	13.5 ± 2.4	13.6 ± 2.9	+0.7
Carbohydrate [%E]	50.7 ± 6.1	50.2 ± 7.1	−1.0
Free sugar [%E]	13.0 ± 5.9	12.8 ± 6.1	+1.5
Sugar sweetened beverage intake [g/1000kcal]	9.9 (0.0; 91.5)	0.0 (0.0; 86.5)	−100
Juice intake [g/1000kcal]	18.4 (0; 61.5)	24.0 (0.0; 75.5)	+30.4
Fruit & vegetable intake [g/1000kcal]	159.6 ± 103.1	158.2 ± 93.7	−0.9
Ultra-processed food intake [g/1000kcal]	296.4 ± 134.4	290.5 ± 121.7	−2.0
Underreported ^3^ [%]	6 (5.6)	14 (13.0)	+233
BMR/TEI	1.4 ± 0.3	1.3 ± 0.3	−7.1
Anthropometric data			
BMI [kg/m²] ^4^	18.2 ± 3.5	18.6 ± 4.0	+2.2
Overweight [%]	16 (14.8)	17 (15.7)	+6.3
Socioeconomic factors [%]			
Maternal overweight ^5^	44 (40.7)		-
Maternal high educational status ^6^	100 (92.6)		-
Maternal employment	94 (87.0)		-
Physical activity ^7^ Low Moderate High	24 (32.9) 25 (34.3) 24 (32.9)	24 (32.9) 24 (32.9) 25 (34.3)	0.0 −4.2 +4.2

Values are frequencies (*n* (%)) or mean ± SD for normally distributed variables or medians (25th; 75th percentile) for non-normally distributed variables. TEI, total energy intake; %E, percentage of total energy intake, BMI, body mass index; BMR, basal metabolic rate. ^1^ One (*n* = 101) or two (*n* = 7) years before the start of the COVID-19 pandemic in March 2020 in Dortmund, Germany. ^2^ during the COVID-19 pandemic in 2020 (15 March–11 August) in Dortmund, Germany. ^3^ Paediatric cut-off values for underreporting [27]. ^4^ BMI cut-off values for children and adolescents [25]. ^5^ BMI > 25 kg/m². ^6^ ≥12 years of schooling. ^7^ tertiles of estimated daily energy expenditure; *n* = 73 (146 observations Pre and current pandemic). Physical activity of the participants was assessed using a standardized questionnaire based on the Adolescent Physical Activity Recall Questionnaires [28] and questions from the German Health Interview and Examination Survey for Children and Adolescents (KiGGS) [29].

**Table 3:** Childcare characteristics of the DONALD participants (subgroup *n* = 71).

Childcare Characteristics	
Females/males [%]	35/65
Age [years]	11.0 (6.7; 15.9)
Type of childcare before pandemic ^1^	
Kindergarten	15 (21.1)
School	52 (73.2)
Others, e.g., family members	4 (5.6)
Childcare visit (school or kindergarten) during pandemic ^1^	
Yes	11 (15.5)
days per week (*n* = 9)	4.0 (3.0; 5.0)
hours per week (*n* = 11)	6.0 (6.0; 7.0)
No	60 (84.5)
Home Office situation during pandemic ^1^	
Mother worked in home office	20 (28.2)
Father worked in home office	13 (18.3)
Both parents worked in home office	21 (29.6)
No parent worked in home office	17 (23.9)

Values are frequencies (*n* (%)) or medians (25th; 75th percentile). ^1^ During the COVID-19 pandemic in 2020 (15 March–11 August) in Dortmund, Germany.

**Table 4:** Dietary intake during the COVID-19 pandemic in 2020 compared to pre-pandemic intake.

	Crude Model		Adjusted Model	
Outcome (Dietary Intake)	β (CI)	*p*	β (CI)	*p*
Difference in total energy intake (kcal) ^1^				
Pandemic	−14.74 (−86.81; 57.32)	0.6859	−109.65 (−187.42; −31.88)	**0.0062**
Pre-pandemic (reference)	0		0	
Difference in fat intake (%E) ^2^				
Pandemic	0.54 (−0.81; 1.90)	0.4280	0.46 (−0.91; 1.83)	0.5038
Pre-pandemic (reference)	0		0	
Difference in protein intake (%E) ^3^				
Pandemic	0.00 (−0.57; 0.57)	0.9930	−0.08 (−0.68; 0.52)	0.7857
Pre-pandemic (reference)	0		0	
Difference in carbohydrate intake (%E) ^2^				
Pandemic	−0.57 (−2.01; 0.88)	0.4409	−0.41 (−1.87; 1.05)	0.5784
Pre-pandemic (reference)	0		0	
Difference in Free sugar intake (%E) ^4^				
Pandemic	−0.20 (−1.34; 0.94)	0.7262	−0.55 (−1.75; 0.66)	0.3685
Pre-pandemic (reference)	0		0	
Difference in Food group intake				
(g/1000 kcal)				
				
Ultra-processed foods ^1^				
Pandemic	−2.89 (−23.41; 17.63)	0.7801	−12.19 (−34.30; 9.93)	0.2764
Pre-pandemic (reference)	0		0	
Fruits and vegetables ^1^				
Pandemic	−1.41 (−17.02; 14.20)	0.8582	2.68 (−13.95; 19.30)	0.7497
Pre-pandemic (reference)	0		0	
Sugar sweetened beverages ^5^				
Pandemic	−2.47 (−21.05; 16.11)	0.7921	−12.78 (−32.16; 6.61)	0.1936 *
Pre-pandemic (reference)	0		0	
Juices ^6^				
Pandemic	7.72 (−5.95; 21.38)	0.2653	8.91 (−4.91; 22.72)	0.2040 *
Pre-pandemic (reference)	0		0	

Comparison of dietary intake during the COVID-19 pandemic in 2020 (15th March–11th August in Dortmund, Germany) and pre-pandemic intake (One (*n* = 101) or two (*n* = 7) years before the start of the COVID-19 pandemic in March 2020) were tested using polynomial mixed-effects regression models; significant p-values of the adjusted models are marked bold; Crude models include predictor (pandemic (yes/no)), outcome (dietary variable) and are adjusted for sex (male/female). ^1^ Model contains a random statement for the family level with an unstructured covariance structure and a random statement for the person level with an unstructured covariance structure. Adjusted models are adjusted for sex (male/female), age (years) and season (summer/autumn/winter/spring). ^2^ Model contains a random statement for the family level with an unstructured covariance structure. Adjusted models are adjusted for sex (male/female) and age (years). ^3^ Model contains a random statement for the family level with an unstructured covariance structure. Adjusted models are adjusted for sex (male/female), maternal employment (yes/no), overweight status of the participant (yes/no), season (summer/autumn/winter/spring) and age (years). ^4^ Model contains a random statement for the family level with an unstructured covariance structure and a repeated statement for the person level with a spatial exponential covariance structure. Adjusted models are adjusted for sex (male/female), season (summer/autumn/winter/spring) and age (years). ^5^ Model contains a random statement for the family level with an unstructured covariance structure. Adjusted models are adjusted for sex (male/female), age (years), maternal high education (yes/no), season (summer/autumn/winter/spring). ^6^ Model contains a random statement for the family level with an unstructured covariance structure and a repeated statement for the person level with an unstructured covariance structure. Adjusted models are adjusted for sex (male/female) and age (years). * Residuals of the final models are not normally distributed.

## Data Availability

Data of the DONALD study is available upon request to epi@uni-bonn.de.

## Supplementary Materials

The following are available online at https://www.mdpi.com/article/10.3390/nu14020297/s1, Table S1: Sensitivity analyses for underreporting.
