# Supplementary material for: Changes in Total Energy, Nutrients and Food Group Intake among Children and Adolescents during the COVID-19 Pandemic—Results of the DONALD Study
Source: Nutrients. 2022 Jan 11;14(2):297. doi: 10.3390/nu14020297 (PMC8778042; doi:10.3390/nu14020297)
Supplement: Supplementary file 1 [file nutrients-14-00297-s001.zip › nutrients-1517067-supplementary.pdf]

Supplementary data

Table S1: Sensitivity analyses for underreporting: Dietary intake during the COVID-19 pandemic in 2020 compared to pre-pandemic intake, excluding participants with underreported records (n=17)

	Crude Model		Adjusted Model	
**Outcome (Dietary intake)**	**β (CI)**	***p***	**β (CI)**	***p***
Difference in total energy intake (kcal)[1]				
Pandemic	20.47 (-42.49; 83.43)	0.5199	-95.77 (-163.68; 27.85)	**0.0063**
Pre-pandemic (reference group)	0		0	
Difference in fat intake (%E)[2]				
Pandemic	0.44 (-1.10; 1.98)	0.5736	0.26 (-1.30; 1.81)	0.7446
Pre-pandemic (reference group)	0		0	
Difference in protein intake (%E)[3]				
Pandemic	0.11 (-0.51; 0.74)	0.7229	0.08 (-0.57; 0.73)	0.8099
Pre-pandemic (reference group)	0		0	
Difference in carbohydrate intake (%E)[2]				
Pandemic	-0.57 (-2.20; 1.05)	0.4833	-0.32 (-1.96; 1.32)	0.6973
Pre-pandemic (reference group)	0		0	
Difference in Free sugar intake (%E)[4]				
Pandemic	-0.00 (-1.25; 1.24)	0.9925	-0.51 (-1.81; 0.80)	0.4442
Pre-pandemic (reference group)	0		0	
Difference in Food group intake (g/1000kcal)				
Ultra processed foods[1]				
Pandemic	4.36 (-16.78; 24.50)	0.6824	-5.54 (28.19; 17.12)	0.6278
Pre-pandemic (reference group)	0		0	
Fruits and vegetables[1]				
Pandemic	-4.52 (-22.05; 13.00)	0.6087	1.92 (-16.76; 20.59)	0.8386
Pre-pandemic (reference group)	0		0	
Sugar sweetened beverages[5]				
Pandemic	0.77 (-17.50; 19.04)	0.9331	-11.25 (-30.16; 7.67)	0.2400*
Pre-pandemic (reference group)	0		0	
Juices[6]				
Pandemic	4.78 (-10.76; 20.31)	0.5426	5.89 (-9.82; 21.59)	0.4583*
Pre-pandemic (reference group)	0		0	

Comparison of dietary intake during the COVID-19 pandemic in 2020 (15th march – 11th august in Dortmund, Germany) and pre-pandemic intake (One (n=85) or two (n=6) years before the start of the COVID-19 pandemic in March 2020) were tested using polynomial mixed-effects regression models; significant p-values of the adjusted models are marked bold; Crude models include predictor (pandemic (yes/no)), outcome (dietary variable) and are adjusted for sex (male/female).[1]Model contains a random statement for the family level with an unstructured covariance structure and a random statement for the person level with an unstructured covariance structure. Adjusted models are adjusted for sex (male/female), age (years) and season (summer/autumn/winter/spring). [2]Model contains a random statement for the family level with an unstructured covariance structure. Adjusted models are adjusted for sex (male/female) and age (years). [3]Model contains a random statement for the family level with an unstructured covariance structure. Adjusted models are adjusted for sex (male/female), maternal employment (yes/no), overweight status of the participant (yes/no), season (summer/autumn/winter/spring) and age (years). [4]Model contains a random statement for the family level with an unstructured covariance structure and a repeated statement for the person level with a spatial exponential covariance structure. Adjusted models are adjusted for sex (male/female), season (summer/autumn/winter/spring) and age (years). [5]Model contains a random statement for the family level with an unstructured covariance structure. Adjusted models are adjusted for sex (male/female), age (years), maternal high education (yes/no), season (summer/autumn/winter/spring). [6]Model contains a random statement for the family level with an unstructured covariance structure and a repeated statement for the person level with an unstructured covariance structure. Adjusted models are adjusted for sex (male/female) and age (years). *Residuals of the final models are not normal distributed
